# Electrocortical N400 Effects of Semantic Satiation

**DOI:** 10.3389/fpsyg.2017.02117

**Published:** 2017-12-05

**Authors:** Kim Ströberg, Lau M. Andersen, Stefan Wiens

**Affiliations:** ^1^Gösta Ekman Laboratory, Department of Psychology, Stockholm University, Stockholm, Sweden; ^2^NatMEG, Department of Clinical Neuroscience, Karolinska Institutet, Stockholm, Sweden

**Keywords:** semantic satiation, N400, priming, ERP, habituation

## Abstract

Semantic satiation is characterised by the subjective and temporary loss of meaning after high repetition of a prime word. To study the nature of this effect, previous electroencephalography (EEG) research recorded the N400, an ERP component that is sensitive to violations of semantic context. The N400 is characterised by a relative negativity to words that are unrelated vs. related to the semantic context. The semantic satiation hypothesis predicts that the N400 should decrease with high repetition. However, previous findings have been inconsistent. Because of these inconsistent findings and the shortcomings of previous research, we used a modified design that minimises confounding effects from non-semantic processes. We recorded 64-channel EEG and analysed the N400 in a semantic priming task in which the primes were repeated 3 or 30 times. Critically, we separated low and high repetition trials and excluded response trials. Further, we varied the physical features (letter case and format) of consecutive primes to minimise confounding effects from perceptual habituation. For centrofrontal electrodes, the N400 was reduced after 30 repetitions (vs. 3 repetitions). Explorative source reconstructions suggested that activity decreased after 30 repetitions in bilateral inferior temporal gyrus, the right posterior section of the superior and middle temporal gyrus, right supramarginal gyrus, bilateral lateral occipital cortex, and bilateral lateral orbitofrontal cortex. These areas overlap broadly with those typically involved in the N400, namely middle temporal gyrus and inferior frontal gyrus. The results support the semantic rather than the perceptual nature of the satiation effect.

## Introduction

The two primary properties of every word are its form (morphology and phonology) and its meaning (semantics and syntax; Levelt, 1989). Separate the two and you are left with a meaningless sound and a nameless concept. This deconstruction of the properties of a word can easily be perceived when hearing someone speak in a language that is foreign to you. Although you may hear the sounds spoken with perfect clarity, you are unable to attribute meaning to them. Conversely it can also occur when speaking a language you do not fully master and attempt to describe a concept only to find that you lack the vocabulary to articulate it. These examples may seem like stating the obvious, yet they serve to exemplify a linguistic dichotomy that is easy to recognise at a distance when applied to foreign languages but much more difficult to perceive in languages we know well. To illustrate, take the example of the word ‘Mother.’ It is difficult to conceptualise the deconstruction of this word in your native tongue – to attempt to completely separate the sounds that make up the word from the semantic concept it represents. Yet this very disassociation appears to be the effect of a commonly experienced but little investigated phenomenon known as semantic satiation, defined as the subjective experience of a temporary loss of the meaning of a word after it has been repeatedly produced or perceived (Balota and Black, 1997). By simply repeating a well-known word enough times its form appears to become disassociated from its meaning.

Subjectively, semantic satiation is often experienced as surprising and amusing, and the satiated word appears to sound strange and meaningless (Esposito and Pelton, 1971; Balota and Black, 1997; Black, 2004). At face value, the effect of this apparent satiation conflicts with a large body of literature in memory and learning that repetition should facilitate processing and retrieval, not inhibit it (for a review, see Crowder, 1976/2014). This pattern, where prolonged activation results in an inhibition of activation, is not unique to semantic satiation, however. Similar effects are well known in cases of sensory habituation and neural adaption whereby constant or repeated perceptual stimuli may appear to fade (see Kohn, 2007, for a review) or be transformed (e.g., Warren, 1968). Because of this, several researchers have questioned the accuracy of referring to the phenomenon as semantic satiation, arguing that the effects are primarily due to perceptual habituation and that the subjective loss of meaning is merely a by-product of this perceptual habituation (Esposito and Pelton, 1971; Frenck-Mestre et al., 1997; Pilotti et al., 1997). According to this view, when a word is repeatedly presented, perceptual habituation occurs. As this increasingly degrading input feeds into semantic memory circuits, retrieval of the semantic association is impeded, resulting in the perceived loss of meaning because of perceptual habituation rather than semantic satiation. According to these models, the satiation is semantic only secondarily, and the phenomenon might be more accurately described as pre-semantic satiation or perceptual satiation. This question regarding the locus of the phenomenon (whether it is primarily semantic or perceptual) has been a key issue in semantic satiation research and an ongoing debate within the field.

Semantic satiation was initially investigated by introspection and subjective reports (Severance and Washburn, 1907; Bassett and Warne, 1919). Later studies used behavioural measures such as reaction time, accuracy, and commonality of associations (for a review, see Esposito and Pelton, 1971). However, in a thorough review of these studies, Esposito and Pelton (1971) concluded that because of the many methodological flaws in these studies, there was in fact no convincing evidence for the existence of a semantic satiation effect. Subsequent research adopted a paradigm known as the lexical decision task in their investigation of the effect (Neely, 1977; Cohene et al., 1978). In a lexical decision task, participants are first presented with a prime word and then required to judge whether the target word presented is real or a pseudo-word. In such a priming paradigm the response time to correctly identify the target as a real word is faster if the preceding prime is semantically related to the target than if they are unrelated. This effect of responding quicker to prime-related targets rather than prime-unrelated targets is referred to as the *relatedness effect*. Because the semantic satiation hypothesis predicts that heavy repetition of primes should lead to their semantic satiation, the relatedness effect should decrease. Yet, in several studies that used the lexical decision task and tested effects of repetition, no such inhibitory effect of repetition was observed (Neely, 1977; Cohene et al., 1978).

In response to this, Smith (1984) argued that the lexical decision task was insensitive to effects of semantic satiation. Because the decision of whether the target is a real word or a pseudo-word can be performed independently of the meaning of the target, this task does not actually require that the target is processed on a semantic level. Furthermore, the processes involved in lexical priming may be different from those in semantic satiation. Therefore, Smith (1984) proposed a category membership task in which participants are primed with a category word (e.g., flower) and instructed to indicate whether the target word (e.g., daffodil) is part of that category or not. This task requires processing of the semantic content of both prime and target, making it sensitive to effects of semantic satiation. When this category membership task was used, the relatedness effect decreased after heavy repetition, providing evidence for a satiation effect (Smith, 1984).

Subsequent studies provided further support for effects of semantic satiation (Smith and Klein, 1990; Balota and Black, 1997; Black, 2001). In several experiments, Balota and Black (1997) found that repeated priming increased response times in a decision task on semantic relatedness. Participants judged the semantic relatedness between a visually presented word-pair after the repeated priming (2, 12, or 22 times) of one of the two words. The relatedness effect was diminished when the prime was repeated 22 times rather than 2 times. Subsequent experiments contributed to clarifying this effect. Specifically, the relatedness effect did not decrease when the heavily repeated prime was unrelated to the word-pair. This finding suggested that the satiation effect was mediated by the semantic relatedness of specific items and not by a general satiation or fatigue of semantic memory. Furthermore, when participants performed a decision task on phonological relatedness (rhyming judgments), high repetition priming did not affect the relatedness effect, supporting the conclusion that the nature of the effects was semantic and not phonological.

Although the findings of Balota and Black (1997) provided evidence for a semantic locus of the effect, several researchers have maintained that the effect is perceptual in nature and results from bottom-up processes of habituation and adaptation (Esposito and Pelton, 1971; Frenck-Mestre et al., 1997; Pilotti et al., 1997). According to this view, the satiation is primarily perceptual and is semantic only secondarily. In support, Pilotti et al. (1997) had participants perform a category membership task like the one used by Smith (1984) but with auditory stimuli. To minimise confounding influences of perceptual processes, Pilotti et al. (1997) varied the physical features of the prime words. In one condition, the auditory stimuli consisted of a single recorded voice, and in the other condition, the auditory stimuli consisted of several different voices. The results showed that satiation occurred in the one-voice condition but not in the many-voice condition. The authors concluded that this pattern of results suggests a perceptual pre-semantic locus of the effect. However, Kounios et al. (2000) argued that the many-voice condition might have induced participants to attend to the acoustic rather than the semantic properties of the stimuli. Such differences in strategy may be particularly likely because Pilotti et al. (1997) used a between-subjects design. Accordingly, because participants in the many-voice condition may have attended less to the semantic properties of the stimuli, the satiation effect might have been eliminated.

Although behavioural measures have been used extensively to study semantic satiation, behavioural responses are only end-state measures that do not permit strong conclusions about the underlying cognitive and neural processes. To examine these processes more directly, two studies have used electroencephalography (EEG) with a focus on the N400. The N400 is characterised by a distinct negativity over central electrodes that occurs between 250 and 500 ms after stimulus onset (Kutas and Federmeier, 2000) to contextually unrelated (vs. related) semantic stimuli. In a landmark study by Kutas and Hillyard (1980), participants were asked to read sentences that were presented one word at a time. In 25% of the trials, the final word in the sentence (e.g., socks) did not fit in with the preceding context (e.g., He spread the warm bread with socks). The N400 corresponded to a relative negativity over central electrodes to final words that were semantically unrelated (vs. related) to the meaning of the preceding words. Many subsequent studies have also found an N400 in semantic priming tasks (e.g., Rugg, 1985; Kutas and Van Petten, 1988; Kutas, 1993). Thus, the N400 may complement behavioural measures and as a direct, selective, and sensitive measure of neocortical activity (Kutas and Federmeier, 2011), it may provide “a window into the neurobiology of meaning” (Kutas and Federmeier, 2000, p. 469).

In the first N400 study of semantic satiation (Frenck-Mestre et al., 1997), participants performed a decision task on category membership. Participants were shown a prime word once and then repeated this prime out loud in rhythm with a visual counter that counted down from either 3 or 30; thus, the prime was repeated either 3 or 30 times. Participants then made a category membership decision in response to the subsequent target word. Overall, the ERPs to the targets showed an effect of prime-target relatedness. Specifically, the ERPs to the targets were more negative when the targets were unrelated than related to the primes, a pattern corresponding to an N400 (for a review, see Kutas and Van Petten, 1988). However, the amplitude of the N400 did not differ depending on whether primes were repeated 3 or 30 times. Since semantic satiation would have predicted a decreased N400 after 30 repetitions, the null effect of repetition is not consistent with such an effect. Similar to Frenck-Mestre et al. (1997), Pilotti et al. (1997) concluded that repetition apparently does not impair the semantic integration of the prime.

Kounios et al. (2000) argued that because the modality was switched between primes (auditory) and targets (visual), the null findings by Frenck-Mestre et al. (1997) do not rule out that semantic satiation might occur if primes and targets are presented within the same modality. To improve the study designs by Frenck-Mestre et al. (1997) and by Pilotti et al. (1997), Kounios et al. (2000) conducted three experiments on the N400 in semantic satiation. In each experiment, a prime word was presented either visually (Experiment 1) or aurally (Experiments 2a and 2b). A target word (related or unrelated) was presented in the same modality as the prime after 1 and 15 repetitions. Importantly, each trial included both low repetition (i.e., 1 repetition) and high repetition (i.e., 15 repetitions) conditions. Unlike previous studies, however, Kounios et al. (2000) did not include a decision task about category membership or relatedness of prime and target. Instead, participants monitored the consecutive words for body parts (e.g., arm, foot) and pushed a button as soon as they saw such a word; these words replaced 4.5% of the primes. This task was to ensure that participants remained attentive and processed the words on a semantic level. Also, to ensure that confounding effects of perceptual habituation were reduced, the repeated primes varied in their physical features. In their first experiment, primes were randomly presented in different letter cases (all lowercase, all uppercase, or only with the primary letter in uppercase). In their last experiment, primes and targets were presented aurally but in a voice in which the frequency of the pitch was altered for repeated primes. In contrast to previous studies (Frenck-Mestre et al., 1997; Pilotti et al., 1997) the main results showed that the N400 between unrelated vs. related primes decreased with repetition, consistent with an effect of semantic satiation. Because these findings were observed despite variations in letter case (Experiment 1) and pitch (Experiment 2b), they suggest that the nature of the effect was semantic and not merely a by-product of perceptual habituation.

Although the results by Kounios et al. (2000) support the idea of semantic satiation, the study design has several limitations. First, because each trial included the target for the low repetition condition (after 1 repetition) and the target for the high repetition condition (after 15 repetitions), this order restriction may have confounded effects of repetition on the N400. For example, the mere presence of the low repetition target before the high repetition target may have decreased the effect of relatedness on the high repetition target for reasons other than repetition. Second, the body-part words always preceded the high repetition targets. As these response trials were not excluded from the analysis, it is possible that processes related to decision making may have confounded the relatedness effect to high repetition targets. Third, although the physical features of the primes were varied across repetition, no details about the randomisation strategy were described.

Because of the limitations of previous studies, the purpose of the present study was to replicate and extend previous research in the visual domain. We used a 2 × 2 repeated-measures design with the variables relatedness and repetition; that is, the targets were either semantically related or unrelated to the primes, and the primes were repeated 3 or 30 times, similar to many previous studies (Smith, 1984; Smith and Klein, 1990; Pynte, 1991; Frenck-Mestre et al., 1997; Pilotti et al., 1997). High and low repetition trials were separated to avoid order confounds. As in the study by Kounios et al. (2000) participants monitored the word stream for items from an irrelevant category. However, because of their simplicity and salience, we used words for colours (red, blue, green and yellow) rather than body parts. We also increased the proportion of trials with these items (from 4.5 to 10%) to enhance participants’ attention to the word stream. Importantly, because response trials with colour words were excluded from the measurement of the N400, any confounding effects from processing and responding to the colour words were minimised. Further, each prime word was created in nine different versions based on the independent combination of letter case (uppercase, lowercase, or initial letter in uppercase) and format (normal, bold, or italic), and consecutive primes always differed on both features. Thus, confounding effects from physical features were minimised. Last, we conducted EEG recordings with 64 electrodes to obtain a comprehensive assessment of the topography of the effects. In sum, we improved on previous research to test for effects of repetition on the N400 in a semantic priming task. Critically, if the N400 is smaller after high repetition than low repetition, this finding of an interaction between relatedness and repetition would provide evidence that the nature of the effect of repetition on relatedness is semantic and not perceptual.

## Materials and Methods

### Participants

Participants were 26 individuals with normal or corrected to normal vision. One participant had to be excluded because of excessive EEG artefacts. The final sample (*N* = 25) comprised 12 women and 13 men (age ranged between 21 and 32 years, *M* = 25.54, *SD* = 2.67). Because all data were collected for the first author’s master’s thesis, we recruited as many participants as possible within a designated interval of 2 months. Participants volunteered to take part in the experiment in exchange for either course credit or a movie voucher. Participants were required to be below the age of 35, to be native Swedish speakers, and to not suffer from any language-based learning disabilities because the experiment included fast-paced reading comprehension. The research was conducted in accordance with the principles of the regional ethics board. Informed, written consent was obtained from all participants.

### Word Stimuli

The word stimuli were generated by the first author and comprised 360 distinct and common Swedish nouns ranging in length between 2 and 12 letters (*M* = 5.41, *SD* = 1.51). Words were shown in size 54 in the sans serif font Arial in the centre of the screen in black on a light grey background. When words were used as primes, they varied in terms of letter case (e.g., häxa, HÄXA, or Häxa, which translates to ‘witch’ in English) and format (normal, bold, or italic). Viewing distance was 57 cm to a 24-inch computer screen with 1920 × 1080 resolution. Thus, word width ranged between 1.3 and 11.5 degrees of visual angle (and word height was about 1 degree of visual angle). The 360 words were grouped into 120 word-triplets. Each word-triplet contained one prime word, one semantically related target word, and one semantically unrelated target word (e.g., one word-triplet was ‘häxa, magi, paraply,’ translating to ‘witch, magic, umbrella’ in English). These lists were constructed in-house. On some trials, a colour name (blue, green, red, or yellow) was shown instead of one of the primes (in the same colour, size, and font as the other nouns).

### EEG Recording

The EEG was recorded with an Active Two BioSemi system (BioSemi, Amsterdam, Netherlands) from 64 Ag/AgCl pin electrodes at standard 10–20 positions and two additional system-specific pin electrodes serving as ground (DRL) and internal reference (CMS) (positioned between POz and either PO3 and PO4, respectively). Electrodes were placed in an EEG-cap (Electro-Cap International, Eaton, OH, United States). Data were sampled at 512 Hz and hardware filtered with a 104 Hz low-pass filter. A Cedrus StimTracker (Cedrus Corporation, San Pedro, CA, United States) was used to detect and mark the onset of the visual stimuli.

### Procedure

**FIGURE 1:**
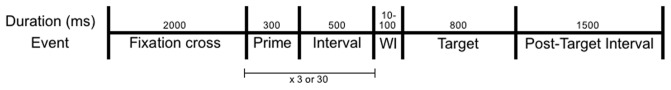
Schematic of each trial with its events and their duration. The prime (300 ms) and the subsequent interval (500 ms) were repeated either 3 or 30 times. *WI* is an additional wait interval between the final prime repetition and the target and was randomised to last between 10 and 100 ms.

Participants were fitted with the EEG-equipment and seated by a table-mounted chin and forehead support. They were instructed to maintain their gaze in the middle of the screen (where individual words were presented), and to silently read each word. To ensure that participants read each word (i.e., attended to the semantic content), their task was to press the space bar whenever a colour word was presented. Each trial was structured as follows (see **Figure 1**): A fixation-cross was presented for 2000 ms and was followed by the repetition phase during which the prime word was repeated either 3 or 30 times. On each repetition, the prime was displayed for 300 ms followed by a 500 ms blank interval. Each prime was shown in one of nine versions, created by combining letter case and format. These versions varied randomly with the restriction that two consecutive repetitions differed on both letter case and format. After the repetition phase, there was a blank wait interval randomised to last between 10 and 100 ms (to reduce synchronisation with alpha waves). After this interval, a target word that was either related or unrelated semantically to the prime was presented for 800 ms. After a blank interval of 1500 ms, the next trial started.

In total, the task consisted of 120 trials. The assignment of the 120 word-triplets was randomised, for each participant, to the two independent variables *repetition* (whether the prime word was repeated 3 or 30 times) and *relatedness* (whether the target word was semantically related to the prime word or not), yielding four separate conditions with 30 trials each. Critically, the random assignment of the word-triplets to the conditions minimised the risk of confounding the effect of repetition on the N400 by physical differences between the word-triplets. Trial order was also randomised, for each participant, with the restriction that the same condition did not occur on more than four consecutive trials. On 12 trials (i.e., 10% of trials, with 3 in each of the four conditions), a colour word was presented. The colour word was chosen randomly from the set of four colour words. On 3 repetition trials, the colour word replaced randomly either the second or third prime, and on 30 repetition trials, the colour word replaced randomly one of the primes in positions 10 through 25. Participants had to press the space bar when they detected this colour word. The experiment lasted for approximately 40 min and was divided into four blocks of 30 trials each. Between blocks, participants were allowed a brief pause. Before the experiment, participants practised the task until they felt comfortable with it.

### ERP Analysis

The EEG data were processed offline with the FieldTrip toolbox in MATLAB (Oostenveld et al., 2010). The continuous data were downsampled to 256 Hz and high-pass filtered at 0.1 Hz with a 4th order infinite impulse response (IIR) Butterworth two-pass filter and -6 dB per octave. On visual inspection of the continuous EEG data, five participants had between two to three noisy electrodes. For each participant, these noisy electrodes were excluded initially and later interpolated after the Independent Component Analysis (ICA) to identify and remove the component that corresponded to eye-blinks. Epochs for ERPs were extracted from 100 ms before target onset to 800 ms after target onset. To minimise any confounding effects from processing and responding to the colour words, epochs were extracted only for the 27 trials in each condition that did not contain colour words (and thus, these trials did not require a motor response). Epochs were baseline corrected relative to the mean EEG activity from 100 ms before target onset to actual target onset. For each participant, amplitude ranges were extracted for each epoch, and the distribution of these amplitude ranges over epochs and electrodes was visually inspected in a summary mode in fieldtrip (ft_rejectvisual) in order to exclude extreme values that could not reasonably be caused by eye-blinks. Importantly, this process was blind to the specific experimental condition of individual trials, as recommended (Keil et al., 2014). For each participant, an ICA was performed to identify and correct for artefacts caused by eye-blinks. After that, any electrodes that were excluded initially (maximum = 3) were spline interpolated, the data were baseline-corrected, and re-referenced to the average across all 64 electrodes. The amplitude ranges over trials were inspected again in a summary mode in fieldtrip (ft_rejectvisual) to exclude remaining outliers. This process was also blind to the specific experimental condition of individual trials. The mean number of trials (of max 27 trials) exceeded 23.6 (*SD* < 2.6) in each of the four conditions; thus, 87% of trials were retained on average for each participant and condition. For the subsequent analyses of the ERP data, the data were re-referenced to electrodes P9 and P10 (close to the common mastoid reference). On the basis of the N400 literature (Kutas and Federmeier, 2000; Kutas and Federmeier, 2011), the N400 was extracted between 250 and 500 ms post-stimulus onset. Originally, we planned to select relevant electrodes by visual inspection of the mean difference ERP between unrelated and related words across repetition. However, when we subsequently viewed the separate topographies of the relatedness effect (i.e., the N400) for each repetition condition, the 3 repetition condition (compared to the 30 repetition condition) showed an N400 that was apparently stronger over centrofrontal than centroposterior electrodes. Further, in the Kounios et al. (2000) study, the effects of repetition on the N400 were strongest for anterior electrodes, as suggested by a three-way interaction between relatedness, repetition, and electrode location (anterior vs. posterior). Therefore, we extracted mean amplitudes from 18 centrally positioned electrodes that were divided into two sets of 9 electrodes, a centrofrontal set (C1, Cz, C2, FC1, FCz, FC2, F1, Fz and F2) and a centroposterior set (CP1, CPz, CP2, P1, Pz, P2, PO3, POz, and PO4). Thus, for each participant, mean amplitudes were computed for these two electrode sets for each of four conditions, created by combining number of repetitions (3 or 30) with relatedness to prime (unrelated or related).

### Cluster Permutation Analysis

As described below, the analyses of these ERPs supported the notion of an effect of repetition on the N400. However, we also followed up these results with a purely data-driven approach for two reasons. First, although the selection of channels (into centrofrontal and centroposterior sets) and of intervals sounds intuitive and was consistent with the literature (Kounios et al., 2000), this selection was not *a priori*. Thus, the reported significant levels may not conform to the strict requirements of confirmatory hypothesis testing. Second, the ERPs for the frontal electrodes suggested that the negativity for unrelated vs. related after 3 repetitions started rather early (after 100 ms).

A general challenge in data-driven ERP analyses is that the data consist of many channels over many time points (e.g., 64 channels during 500 ms at 250 Hz gives 64 × 125 = 8000 data points). If a significance test is performed for each combination of channel by time point, this mass univariate analysis creates a multiple comparison problem: with numerous comparisons, some will be significant just by chance (Groppe et al., 2011a). One approach is to conduct Bonferroni correction, but this approach is very conservative and has low power (Groppe et al., 2011b). A more sensitive approach is cluster permutation analysis (Maris and Oostenveld, 2007; Maris, 2012). This approach takes advantage of the fact that genuine ERP effects should not be localised to individual data points but should involve many channels that are activated over several time points. The analysis groups together data points that are neighbours in space and in time into clusters and computes the probability to observe these clusters by chance. Critically, even if one or more clusters have low probability given chance (e.g., *p* < 0.05), this does not mean that the activation can be localised to the channels and time points of the particular clusters. The analysis supports only the conclusion that there are differences between the conditions (i.e., the data do not come from the same probability distribution). Accordingly, the channels and time points of the clusters should be viewed only as suggestive evidence for where and when the main activation may have occurred. However, focusing on the clusters makes sense because the occurrence of clusters is physiologically plausible (van Ede and Maris, 2016).

For the cluster permutation analysis and the subsequent source reconstruction, we re-referenced the data to the arithmetic average across all electrodes and applied a 30-Hz low-pass filter (two-pass Butterworth, 4th order). The cluster permutation mainly used the default settings that are supplied by Fieldtrip. The analysis included each combination of channel and time point in the 100 to 600 ms interval after word onset. For a given analysis between conditions (e.g., unrelated after 3 repetitions vs. related after 3 repetitions), individual data points were considered to be included in a cluster if they were significant by themselves in an uncorrected paired-samples *t*-test (alpha = 0.05, two-tailed). Data points with negative or positive activity were grouped together separately. To minimise sensitivity to tiny clusters, which are physiologically implausible, data points were included in a cluster only if they also had at least two neighbours (as determined by triangulation) that were each significant in the individual *t*-test. For each cluster, the *t*-values for these data points were summed together. Finally, from these sums of *t*-values, the maximum over clusters was recorded. In the actual permutation test, the condition labels were randomly assigned to the data (5000 repetitions). For each repetition, clusters were formed, and from the sum of *t*-values for each cluster, the maximum over clusters was recorded. From the 5000 repetitions, a distribution of these maximum sums of *t*-values was generated and used to compute the probability (*p*) of the observed maximum (or even larger maxima). We considered clusters if the analysis showed *p* < 0.05 (one-tailed). Because negative and positive clusters were tested separately, this threshold corresponds to α = 0.10 (two-tailed). Although a two-tailed α = 0.05 is more conventional, the one-tailed threshold reflects our one-directional prediction (i.e., the negativity of the N400) and also improves sensitivity in detecting an effect.

### Source Reconstruction

To explore the cortical sources giving rise to the scalp topographies, we used the sLORETA algorithm (Pascual-Marqui, 2002) to estimate the currents of underlying sources. Because no individual magnetic resonance images were available, a template brain (Tzourio-Mazoyer et al., 2002) was used, both for modelling the cortex (sources 10 mm apart) and the volume conductor (a boundary element method model, modelling the brain, the skull and the skin separately with unique conductivities). Source reconstruction was performed on the mean ERP amplitudes in the intervals that were identified in the cluster permutation analysis. For each test, sources were considered if their activity was either in the minimum or maximum range (i.e., in the lower or upper 2.5% of the activity distributions). Because this analysis was explorative with the purpose to identify the most likely sources for the observed scalp effects, no significance testing was performed.

## Results

All stimuli, scripts, raw and preprocessed data, and results are available online (via figshare at this doi: 10.17045/sthlmuni.5468455). In the analyses below, we chose to conduct contrast *t*-tests (Wiens and Nilsson, 2016). Although readers may be more familiar with ANOVAs and *F* tests, the advantages of *t*-tests are that they are specific and directional compared to *F* tests. Notably, for any of the *t*-tests below that could be performed as *F* tests, the results of these *F* tests would give identical *p*-values with *F* = *t*^2^ because the *F* tests have only 1 *df* in the numerator (Rosenthal et al., 1999, *p*. 3). The *p*-values and 95 confidence intervals (95% CIs) reported below are uncorrected.

### Behavioural Data

Performance in responding to the colour words served to ensure that attention was maintained and items processed on a semantic level. A colour word was considered as detected if the response occurred within 200–1000 ms after onset of the colour word (i.e., before the response may have actually occurred to the next prime word). Across participants, mean number of correctly detected colour words was 10.48 out of 12 trials (*SD* = 2.18). False alarms were any responses during a trial without a colour word (the possible maximum was 4 × 27 = 108). Across conditions, the number of false alarms was low (*M* = 1.12, *SD* = 1.67).

### ERP Data

**FIGURE 2:**
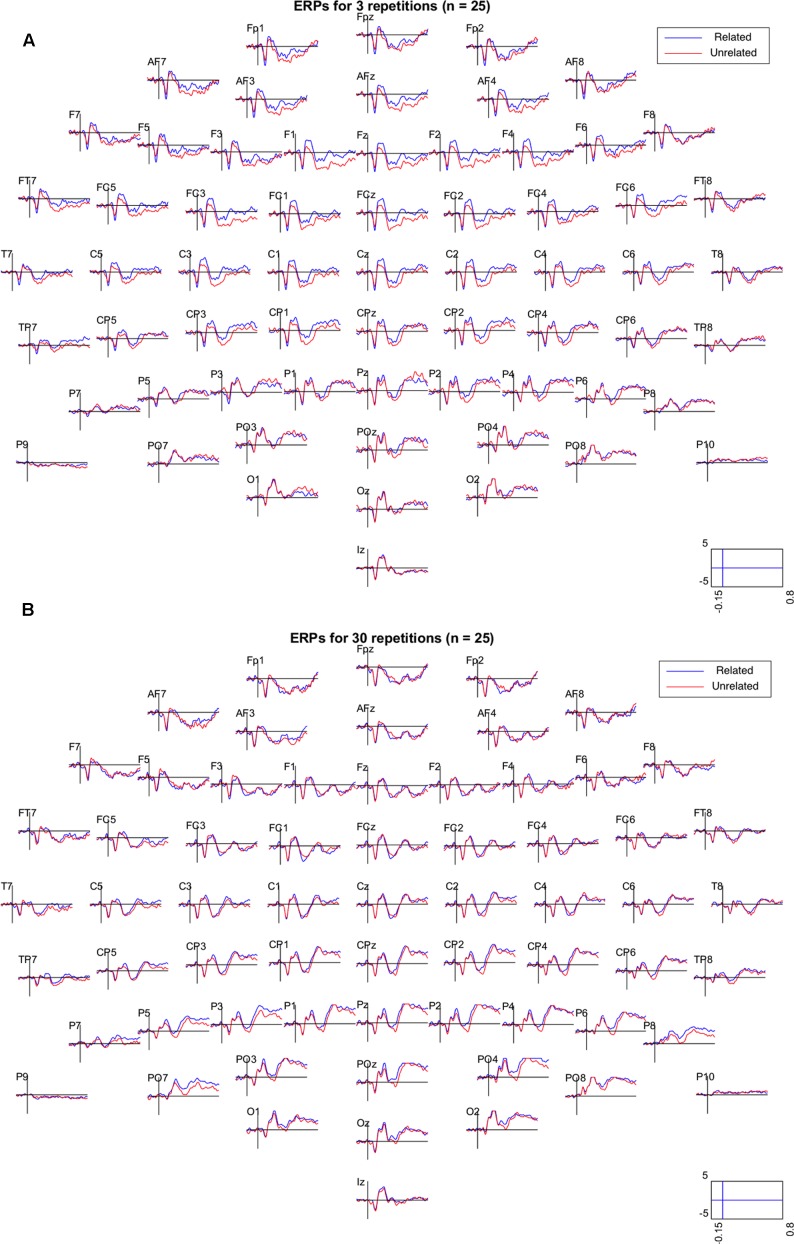
Grand mean ERPs (*N* = 25) from all 64 electrodes for related (blue) and unrelated (red) target items in the 3 repetition condition **(A)** and the 30 repetition condition **(B)**. Electrodes were referenced to the mean of electrodes P9 and P10 (closest to mastoids). Activity is shown in μV between –0.15 and 0.8 s relative to target onset. In the plots, the data were low-pass filtered at 30 Hz.

**FIGURE 3:**
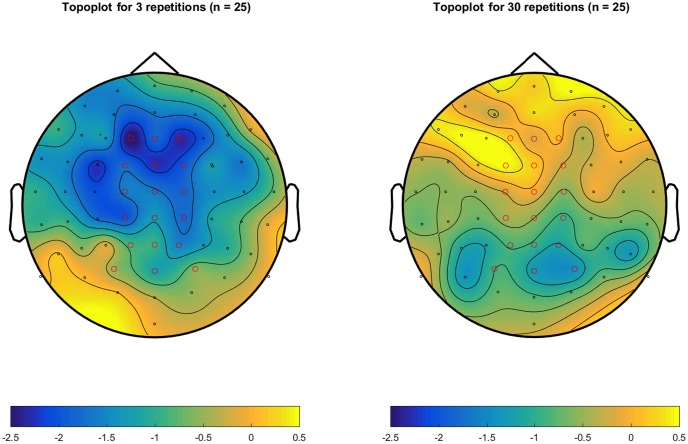
Topoplots of the mean amplitude differences (in μV) between unrelated and related targets across 250–500 ms after target onset, separately for the 3-repetititon condition (Left) and the 30 repetition condition (Right). Electrodes were referenced to the mean of electrodes P9 and P10 (closest to mastoids). Electrodes in red mark the centrofrontal electrodes (C1, Cz, C2, FC1, FCz, FC2, F1, Fz, and F2) and the centroposterior electrodes (CP1, CPz, CP2, P1, Pz, P2, PO3, POz, and PO4) used to compute mean amplitudes.

**Figure 2** shows the grand mean ERPs elicited by related and unrelated target words from each of the 64 electrodes, separately for the 3 repetition and the 30 repetition condition. In the 3 repetition condition (**Figure 2A**), waveforms associated with unrelated targets are visibly more negative than those associated with related targets, particularly in the 250–500 ms range over centrofrontal electrodes. In the 30 repetition condition, this difference between related and unrelated targets is not readily apparent. In support, **Figure 3** shows the topographies of the relatedness effect (i.e., unrelated minus related) in the 250–500 ms after target onset, separately for the 3 repetition condition (left) and the 30 repetition condition (right). Compared to the 30 repetition condition, the 3 repetition condition showed a greater negativity to unrelated than related targets (i.e., an N400) in centrofrontal electrodes.

**FIGURE 4:**
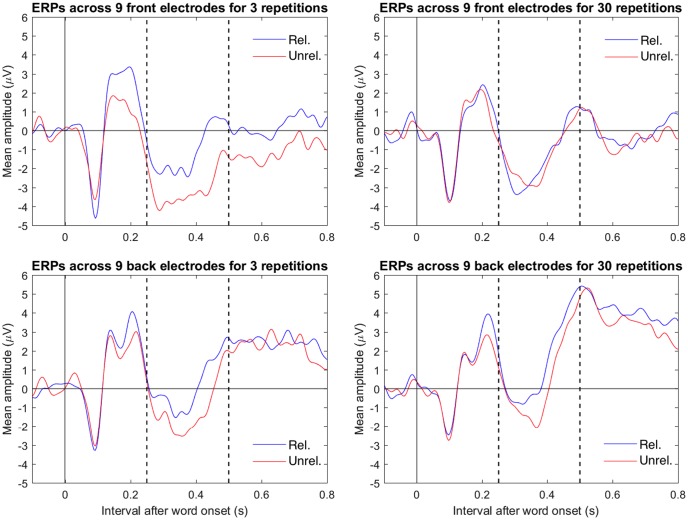
Combined mean ERPs of related (blue) and unrelated (red) trials divided by location (top row shows centrofrontal electrodes C1, Cz, C2, FC1, FCz, FC2, F1, Fz, and F2 and the bottom row shows centroposterior electrodes CP1, CPz, CP2, P1, Pz, P2, PO3, POz, and PO4) and repetition (left column shows 3 repetitions and right column shows 30 repetitions). Electrodes were referenced to the mean of electrodes P9 and P10 (closest to mastoids). In the plots, the data were low-pass filtered at 30 Hz. The dotted vertical lines mark the interval (between 250 and 500 ms after target onset) that defined the N400-relevant interval in the analysis.

**Figure 4** shows the mean ERPs to related and unrelated target words at 9 centrofrontal (C1, Cz, C2, FC1, FCz, FC2, F1, Fz and F2) and 9 centroposterior (CP1, CPz, CP2, P1, Pz, P2, PO3, POz and PO4) electrodes, divided by repetition. A contrast analysis performed as a *t*-test (Wiens and Nilsson, 2016) showed evidence for a three-way interaction between repetitions (3 minus 30), relatedness (unrelated minus related), and electrode location (centrofrontal minus centroposterior). The mean difference for this contrast was -1.69 μV, *t*(24) = -3.61, *p* = 0.001, 95% CI [-2.65, -0.72]. This finding supports the conclusion from **Figures 2** and **3**, namely that the relative negativity for unrelated vs. related target words (i.e., the N400) was larger in the 3 repetition condition than in the 30 repetition condition in centrofrontal electrodes. Because of this three-way interaction, results were further examined separately for each electrode location (centrofrontal and centroposterior).

For the centrofrontal electrodes, there was a two-way interaction between repetition (3 minus 30) and relatedness (unrelated minus related); the mean difference was -1.95 μV, *t*(24) = 2.29, *p* = 0.031, 95% CI [-3.72, -0.19]. Further, only the 3 repetition condition showed a relative negativity (i.e., an N400) in mean amplitudes between unrelated targets (*M* = -3.11, *SD* = 5.87) and related targets (*M* = -1.13, *SD* = 5.73). The mean difference was -1.99 μV, *t*(24) = 2.48, *p* = 0.020, 95% CI [-3.64, -0.34]. The 30 repetition condition did not show such a relative negativity between unrelated targets (*M* = -1.47, *SD* = 6.45) and related targets (*M* = -1.44, *SD* = 5.87). The mean was -0.04 μV, *t*(24) = 0.08, *p* = 0.941, 95% CI [-1.01, 0.94].

For the centroposterior electrodes, there was no apparent two-way interaction between repetition (3 minus 30) and relatedness (unrelated minus related); the mean difference was -0.27 μV, *t*(24) = -0.47, *p* = 0.643, 95% CI [-1.45, 0.91]. Across repetition conditions, there was an N400, as shown by a relative negativity between unrelated and related targets. The mean was -1.09 μV, *t*(24) = -2.86, *p* = 0.009, 95% CI [-1.87, -0.30].

The main finding was that for centrofrontal electrodes, the relative negativity between unrelated and related targets (i.e., the N400) decreased from the 3 repetition condition to the 30 repetition condition (*M* = -1.95). Although the significant *p*-value in this analysis (*p* = 0.031) provided evidence against the null hypothesis, the *p*-value does not necessarily provide evidence for the alternative hypothesis (Dienes, 2016; Wagenmakers et al., 2016). For example, the mean effect of -2 may not actually support a theory that predicts a difference of -4, as the observed effect would fall halfway between 0 and -4 (Dienes and Mclatchie, 2017). Therefore, we conducted Bayesian hypothesis testing to determine the degree of evidence for the alternative hypothesis (Dienes, 2008, 2016). We computed a Bayes Factor (BF_10_) that expresses the likelihood of the data given the alternative hypothesis (i.e., predictions from semantic satiation) relative to the likelihood of the data given the null hypothesis (Dienes, 2008, 2016; Wiens and Nilsson, 2016). The alternative hypothesis needs to capture the idea that the N400 decreases with repetition; that is, the N400 in the 3 repetition condition should decrease in the 30 repetition condition. To estimate the size of this effect of semantic satiation, the 3 repetition condition can be viewed as the baseline condition, and whatever the size of the N400 in this condition, the 30 repetition condition could have no stronger effect than to eliminate the N400 observed in the 3 repetition condition. Accordingly, the size of the N400 in the 3 repetition condition defines a reasonable upper limit for the effect of semantic satiation on the N400 in the 30 repetition condition. In the present study, the N400 was -1.99 μV for centrofrontal electrodes in the 3 repetition condition. Notably, visual inspection of the results of Experiment 1 by Kounios et al. (2000) suggested that the maximum N400 (as seen in the low repetition condition between related and unrelated targets) was also approximately -2 μV at Fz and Cz. Taken together, these findings suggest that the maximum expected change in the N400 from the 3 repetition condition to the 30 repetition condition should be 2 μV (a positive difference score is obtained by subtracting the N400 to the 3 repetition condition from the N400 to the 30 repetition condition). This value of 2 μV was used to define the alternative hypothesis. Critically, this definition is independent from the data in the 30 repetition condition and thus, the BF_10_ is not biased (Dienes, 2014, 2016).

**FIGURE 5:**
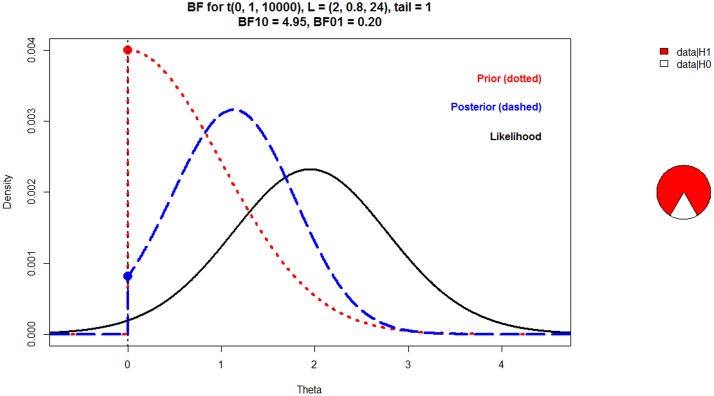
Plot of the prior, likelihood, and posterior on mean amplitudes for the interaction between relatedness and repetition for centrofrontal electrodes. To capture the interaction, the N400 in the 3 repetition condition was subtracted from the N400 in the 30 repetition condition. The positive difference score (*M* = 1.95) suggested that the N400 decreased from the 3 repetition condition (i.e., a large negative number) to the 30 repetition condition (i.e., a small negative number). Theta is the hypothetical effect size (in μV). The Bayes Factor (BF_10_) is illustrated in the pie chart. The larger the red area, the more likely the data are given the alternative hypothesis (semantic satiation) rather than the null hypothesis (of no effect). The alternative hypothesis was expressed as a half-normal distribution to capture the idea that the maximum expected effect size is 2 μV but probably smaller. The BF = 4.95 means that the data support semantic satiation five times more than no effect.

The BF_10_ was calculated with *Aladins Bayes Factor in R* (Wiens, 2017). These scripts compute and plot the BF_10_ for mean differences in raw units if the alternative hypothesis and/or the likelihood are modelled as normal, *t* or uniform distributions (Dienes and Mclatchie, 2017). As described above, for centrofrontal electrodes, the N400 changed by 1.95 μV (i.e., N400 in 30 repetition condition minus N400 in 3 repetition condition), *t*(24) = 2.29. For any *t*-test, *t* = mean/*SEM* (Dienes, 2014). Accordingly, *SEM* = mean/*t* = 1.95/2.29 = 0.85. Therefore, the likelihood of the present data can be modelled as a *t* distribution centred on 1.95 with *SEM* = 0.85 and *df* = 24. Assuming a maximum expected change of 2 μV (as explained above), the alternative hypothesis was modelled as a half-normal distribution with a standard deviation = 1. This alternative hypothesis expresses the idea that the reasonable maximum expected change is 2 μV (i.e., 95% of the weight is below 2 μV) but that smaller values are more plausible. Thus, the mean of the true effect size distribution was set at 0 with a standard deviation of 1 (i.e., half of the maximum effect size of 2). **Figure 5** shows the *prior* (i.e., the alternative hypothesis), *likelihood*, and *posterior*, and also a pie chart to illustrate the obtained BF_10_ = 4.95. The data are about five times more likely given the alternative hypothesis than given the null hypothesis. The BF_10_ is also illustrated by the pie chart: the larger the red area, the more likely the data are given the alternative hypothesis (Wagenmakers et al., 2016). To examine the robustness of the results (Dienes, 2014), the alternative hypothesis was also modelled as a uniform and a normal distribution. Specifically, a *uniform* model captured the notion that the true effect may range uniformly between 0 and 2; the BF_10_ was 6.52. A *normal* model captured the notion that the true effect is normally distributed around half the maximum effect size. Thus, the mean of the true effect size distribution was set at 1 with a standard deviation of 0.5; the BF_10_ was 6.45. These findings support the robustness of the results. Irrespective of the model of the alternative hypothesis, the data supported the alternative hypothesis at least five times more than the null hypothesis. Although the Bayes Factor is a continuous measure of evidence, a BF_10_ > 3 is typically viewed as moderate evidence for the alternative hypothesis, as it tends to match a *p* = 0.05 (Dienes, 2016; Wagenmakers et al., 2017).

### Cluster Permutation Analysis

For the difference between unrelated minus related in the 30 repetition condition, the analysis suggested no evidence for an effect (*p* > 0.33). In contrast, for the difference between unrelated minus related in the 3 repetition condition, the analysis suggested one negative cluster over 11 frontal electrodes between 433 and 489 ms. Although this negativity tended to start earlier and was rather long lasting (as suggested in **Figure 4**), this negativity was strongest between about 400 and 500 ms (see Supplementary Material). Next, we performed a cluster permutation analysis of the interaction, that is, of the difference between unrelated minus related in the 3 repetition condition minus the difference between unrelated minus related in the 30 repetition condition. The cluster analysis suggested two negative clusters involving frontal electrodes between 265 and 325 ms and between 397 and 465 ms (see Supplementary Material).

### Source Reconstruction

**FIGURE 6:**
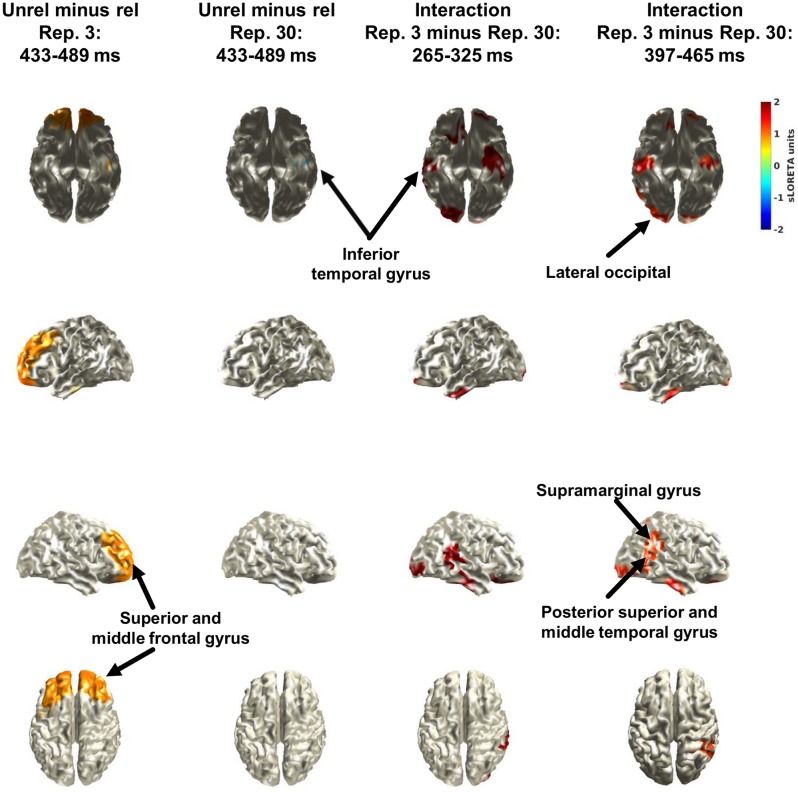
Results of the source reconstruction with sLORETA. The left two columns show the effect of relatedness (unrelated minus related) separately for the 3 repetition condition and the 30 repetition condition for the interval (433–489 ms) that was suggested in the cluster permutation analysis of only the 3 repetition condition (i.e., simple effect). The two right columns show the effect of the interaction between relatedness (unrelated minus related) and repetition (3 minus 30) for the two intervals that were suggested in the cluster permutation analysis of the interaction. Only the minima and maxima (i.e., 2.5% in each tail) are colour coded. These were derived from pooling separately the distributions of the simple effects (i.e., the two left columns) and of the interactions (i.e., the two right columns).

**Figure 6** shows the results of the source reconstruction. Only the minima and maxima (i.e., 2.5% in each tail) are colour coded. These were derived from pooling separately the distributions of the simple effects (i.e., the two left columns) and of the interactions (i.e., the two right columns). Because the cluster permutation analysis suggested an effect of relatedness (unrelated minus related) between 433 and 489 ms when considering only the 3 repetition condition, the two left columns show the results for the effect of relatedness at this interval, separately for the 3 repetition condition and for the 30 repetition condition. In the 3 repetition condition, relatedness increased activity in bilateral superior and middle frontal gyrus and left inferior temporal gyrus. In the 30 repetition condition, relatedness decreased activity in left inferior temporal gyrus. As the cluster permutation analysis suggested an interaction between relatedness and repetition (3 minus 30 repetitions) for the intervals between 265 and 325 ms and between 397 and 465 ms, the two right columns show the source reconstructions of the interaction for these intervals. As shown, the sources of activity overlapped broadly and included bilateral inferior temporal gyrus, the right posterior section of the superior and middle temporal gyrus, right supramarginal gyrus, bilateral lateral occipital cortex, and bilateral lateral orbitofrontal cortex.

## Discussion

The main finding was that the N400 over centrofrontal electrodes decreased from the 3 repetition condition to the 30 repetition condition. Explorative source reconstructions suggested that activity decreased from the 3 repetition to the 30 repetition condition in bilateral inferior temporal gyrus, the right posterior section of the superior and middle temporal gyrus, right supramarginal gyrus, bilateral lateral occipital cortex, and bilateral lateral orbitofrontal cortex.

In the main previous study by Kounios et al. (2000), each high repetition trial included also a low repetition trial. In the present study, the different conditions were tested in separate trials to avoid confounds from this order restriction. Further, participants monitored the semantic content of each word and responded to colour words. These trials were excluded to avoid confounds from response-related processing of the colour words. Furthermore, consecutive prime words varied in both letter case and format to minimise confounds from repeating the same physical features (i.e., perceptual habituation). Therefore, the decrease of the N400 from the 3 repetition condition to the 30 repetition condition is unlikely to be caused by any of these potential confounds.

Although the precise process represented by the N400 is unknown (for a review, see Lau et al., 2008), the N400 is known for its remarkable sensitivity to the semantic context of stimuli (Kutas and Federmeier, 2011). In the context of a typical semantic priming paradigm, the N400 reflects perceived violations of semantic context, as reflected by a relative negativity to words that are unrelated vs. related to the semantic context. In the present study, there was a relative negativity to target words that were unrelated rather than related to the preceding prime words in the 3 repetition condition. This was supported by analyses of mean amplitudes (see **Figure 4**) and the more data-driven cluster permutation analysis (at about 450 ms). These findings match that of an N400 and are a good manipulation check because they replicate many previous findings of the N400 in semantic priming (e.g., Rugg, 1985; Kutas and Van Petten, 1988; Kutas, 1993; Frenck-Mestre et al., 1997; Kounios et al., 2000).

The semantic satiation hypothesis predicts a decrease of the N400 with repetition (Frenck-Mestre et al., 1997; Kounios et al., 2000). If a prime word is sufficiently repeated until it loses its semantic meaning, it will also lose its means of establishing a semantic context for the target. Because the effect of semantic relatedness between prime and target would be weakened, the N400 should decrease. In this study, we computed the Bayes Factor to measure the strength of evidence for the semantic satiation hypothesis relative to the null hypothesis. We modelled semantic satiation in different ways to assess the robustness of our conclusions (Dienes, 2016). In all cases, the Bayes Factor was above 5. This means that the present results support the semantic satiation hypothesis five times more than the null hypothesis (of no effect of repetition). Accordingly, the present finding that the N400 over centrofrontal electrodes decreased from the 3 repetition condition to the 30 repetition condition provides support for the hypothesis of semantic satiation.

The present N400 findings replicate the findings of Kounios et al. (2000) but differ from those of Frenck-Mestre et al. (1997) whose results did not indicate an interaction effect of relatedness and repetition. In light of the present results as well as those of Kounios et al. (2000), this strengthens Kounios et al.’s (2000) suggestion that the lack of an interaction effect in Frenck-Mestre et al. (1997) may be the result of the modality change present in their design, shifting from auditory to visual stimuli between prime and target.

One of the main design features of this experiment was that it reduced the potentially confounding effects of perceptual habituation. To investigate whether the effects of satiation would be observed even if the visual characteristics of the primes varied over repetitions, the present study used nine distinct visual variations of the prime words that were fully randomised so that no two consecutive repetitions were alike on either of two parameters (letter case and format). Accordingly, it is unlikely that the present results were caused by perceptual habituation. However, the current study did not explicitly control for the potential influence of phonological or orthographic processing. Because these are known to influence the N400 (Kutas et al., 2007), the present findings do not eliminate the possibility of a potential influence of orthographic and phonological factors. Hence, future studies should address these issues. Nonetheless, in light of the well-documented findings that the N400 varies systematically with the processing of semantic information, we argue that it seems most plausible that the nature of the effect is semantic rather than orthographic or phonological.

One noteworthy aspect of our study was that the interaction effect between repetition and relatedness was only observed in centrofrontal electrodes whereas centroposterior electrodes showed a similar N400 across both repetition conditions. Notably, this three-way interaction effect of relatedness, repetition, and electrode location was similar to that observed by Kounios et al. (2000). On the one hand, the topography of a centrofrontal distribution of the N400 and the repetition effect is somewhat unexpected because the scalp distribution of the N400 is most often cited as being primarily central and parietal in its topography (Duncan et al., 2009). On the other hand, the topography of the N400 is known to have a widespread scalp distribution that can be difficult to pinpoint (Duncan et al., 2009; Kutas and Federmeier, 2011). Future studies should pre-register their design, electrode locations, and intervals to provide convincing evidence in regards to the robustness of the centrofrontal topography.

Because the selection of electrodes and intervals for the interaction was not entirely *a priori*, we also conducted data-driven cluster permutation analyses together with explorative source reconstructions. The cluster analyses suggested two frontal clusters between 265 and 325 ms and between 397 and 465 ms. The topographies of the two clusters were similar, but the underlying ERP waves were rather noisy (see Supplementary Material). Thus, it seems reasonable to conclude that the findings support a single effect on the N400, but that this effect waxed and waned because of noise. In support of this notion for a similar effect in both clusters, explorative source reconstructions suggested similar areas (**Figure 5**). Specifically, activity decreased from the 3 repetition to the 30 repetition condition in bilateral inferior temporal gyrus, the right posterior section of the superior and middle temporal gyrus, right supramarginal gyrus, bilateral lateral occipital cortex, and bilateral lateral orbitofrontal cortex. These areas overlap broadly with those typically involved in the N400, namely middle temporal gyrus and inferior frontal gyrus (Lau et al., 2008). The results of the source reconstructions are encouraging, given that only a template brain was used because no individual magnetic resonance images were available.

One limitation of the experiment is that the item list was previously untested since it was constructed in-house for the specific purpose of the present experiment. As a result, the specific degree of semantic relatedness between primes and their related and unrelated targets are unknown. However, great care was taken in its design to ensure an intuitive separation between related and unrelated targets that could reasonably be assumed to correspond to the perception of the general population. Furthermore, credible measures of the lexical frequency of the items could not be obtained. As such, the level of familiarity of the words included in the experiment were subjectively established, but again great care was taken to ensure that the items would be common and familiar to native speakers.

Another concern may be the low number of trials (about 27 trials) per condition. This was to keep the experiment under 1 h. For the N400, Luck (2014) suggests 30 to 40 trials for a sample size of 16 participants. Importantly, our sample is large (*N* = 25) and may thus compensate for the noise within subjects. Second, Luck (2014) also discusses that fewer trials increase noise within subjects but that mean amplitude (vs. peak amplitude), as extracted here, is an unbiased measure. Finally, the data speak for themselves, as the clean and clear ERPs in each of the four conditions (as seen in **Figures 2**, **4**) support the conclusion that the number of trials was not an issue.

To our knowledge, the present study is only the third study on the effects of semantic satiation on the N400 (Frenck-Mestre et al., 1997; Kounios et al., 2000). The experiment showed that repetition decreases the N400, as hypothesised by the semantic satiation hypothesis. Furthermore, the results suggest that semantic satiation is not caused primarily by perceptual factors but instead appears to have a semantic locus. In light of these findings the phenomenon of semantic satiation may potentially reveal a great deal about the link between form and meaning in a language, and the questions of how and why this phenomenon occurs are of great interest. To truly understand the neuropsychology of meaning, it is important to also understand the loss of meaning, and as such, semantic satiation could provide valuable insight.

## Ethics Statement

This study was carried out in accordance with the recommendations of the Central Ethical Review Board in Sweden with written informed consent from all subjects. All subjects gave written informed consent in accordance with the Declaration of Helsinki.

## Author Contributions

KS and SW designed the study. KS collected the data. KS and SW analysed the data. LMA contributed the source reconstructions. All authors wrote the final manuscript.

## Conflict of Interest Statement

The authors declare that the research was conducted in the absence of any commercial or financial relationships that could be construed as a potential conflict of interest.
