# Supplementary material for: Electrocortical N400 Effects of Semantic Satiation
Source: Front Psychol. 2017 Dec 5;8:2117. doi: 10.3389/fpsyg.2017.02117 (PMC5770629; doi:10.3389/fpsyg.2017.02117)
Supplement: Supplementary file 1 [file Presentation_1.PPTX]

## Slide 1
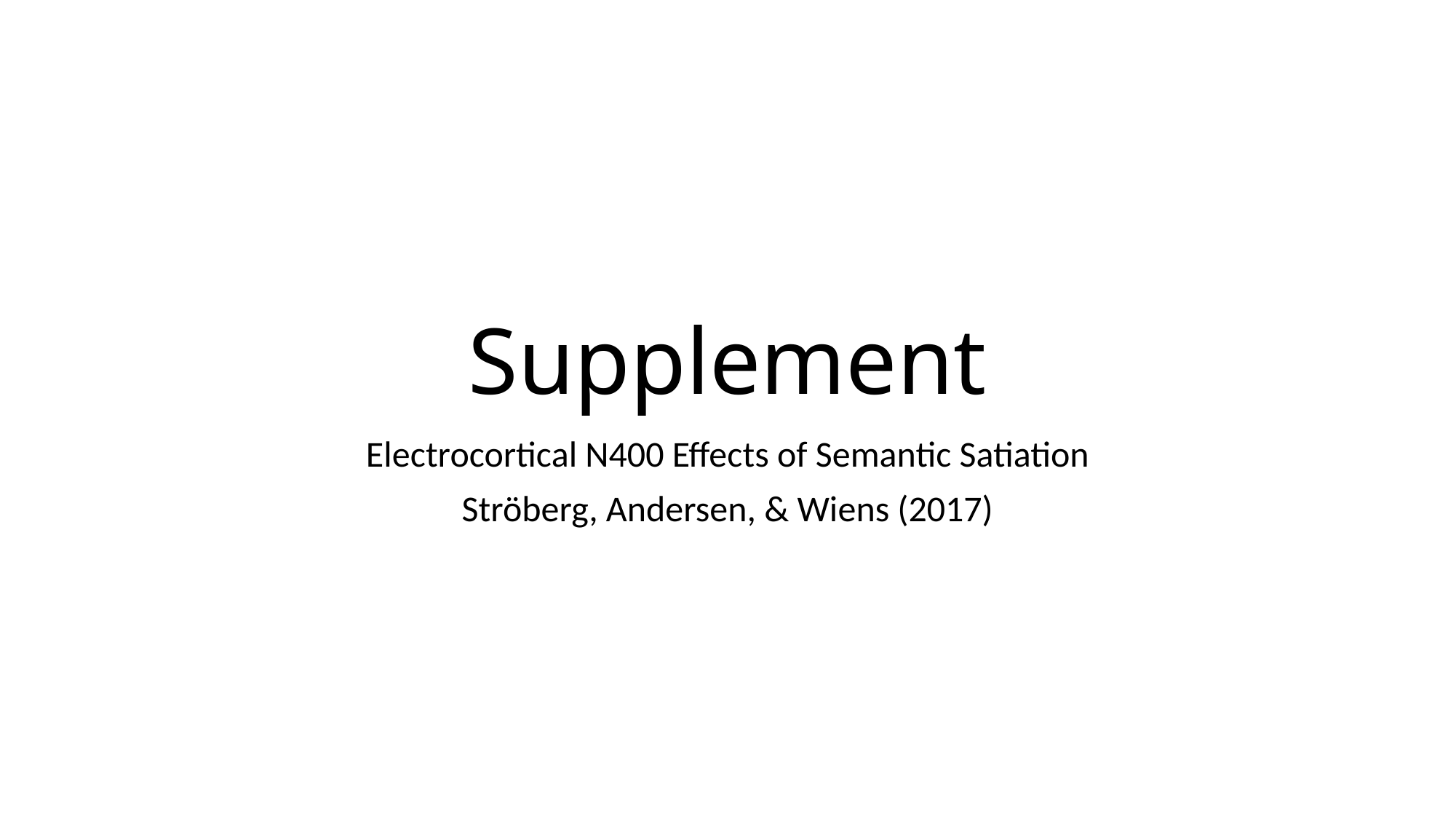

# Supplement
Electrocortical N400 Effects of Semantic Satiation
Ströberg, Andersen, & Wiens (2017)

## Slide 2
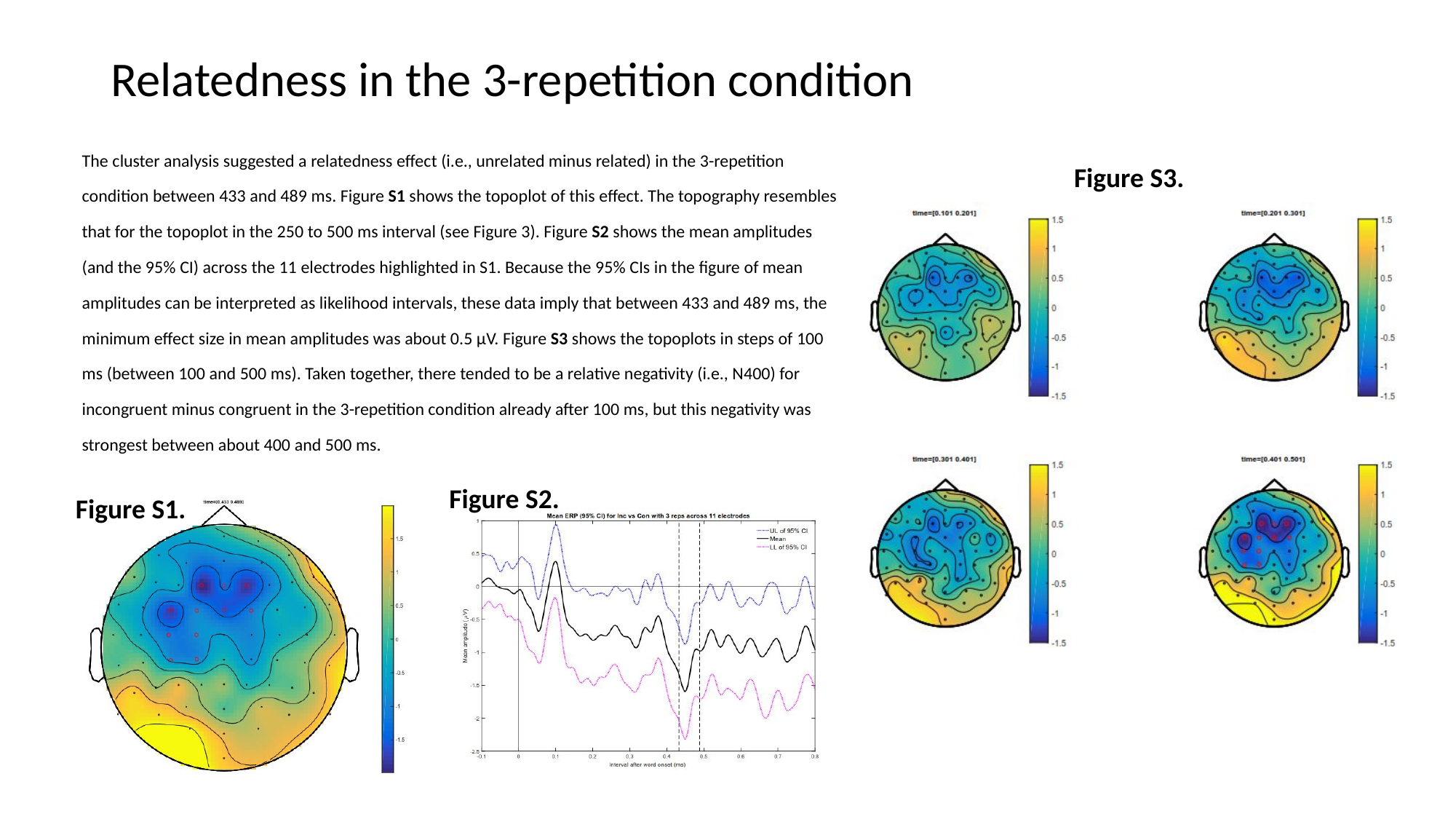

# Relatedness in the 3-repetition condition
The cluster analysis suggested a relatedness effect (i.e., unrelated minus related) in the 3-repetition condition between 433 and 489 ms. Figure S1 shows the topoplot of this effect. The topography resembles that for the topoplot in the 250 to 500 ms interval (see Figure 3). Figure S2 shows the mean amplitudes (and the 95% CI) across the 11 electrodes highlighted in S1. Because the 95% CIs in the figure of mean amplitudes can be interpreted as likelihood intervals, these data imply that between 433 and 489 ms, the minimum effect size in mean amplitudes was about 0.5 µV. Figure S3 shows the topoplots in steps of 100 ms (between 100 and 500 ms). Taken together, there tended to be a relative negativity (i.e., N400) for incongruent minus congruent in the 3-repetition condition already after 100 ms, but this negativity was strongest between about 400 and 500 ms.
Figure S3.
Figure S2.
Figure S1.

## Slide 3
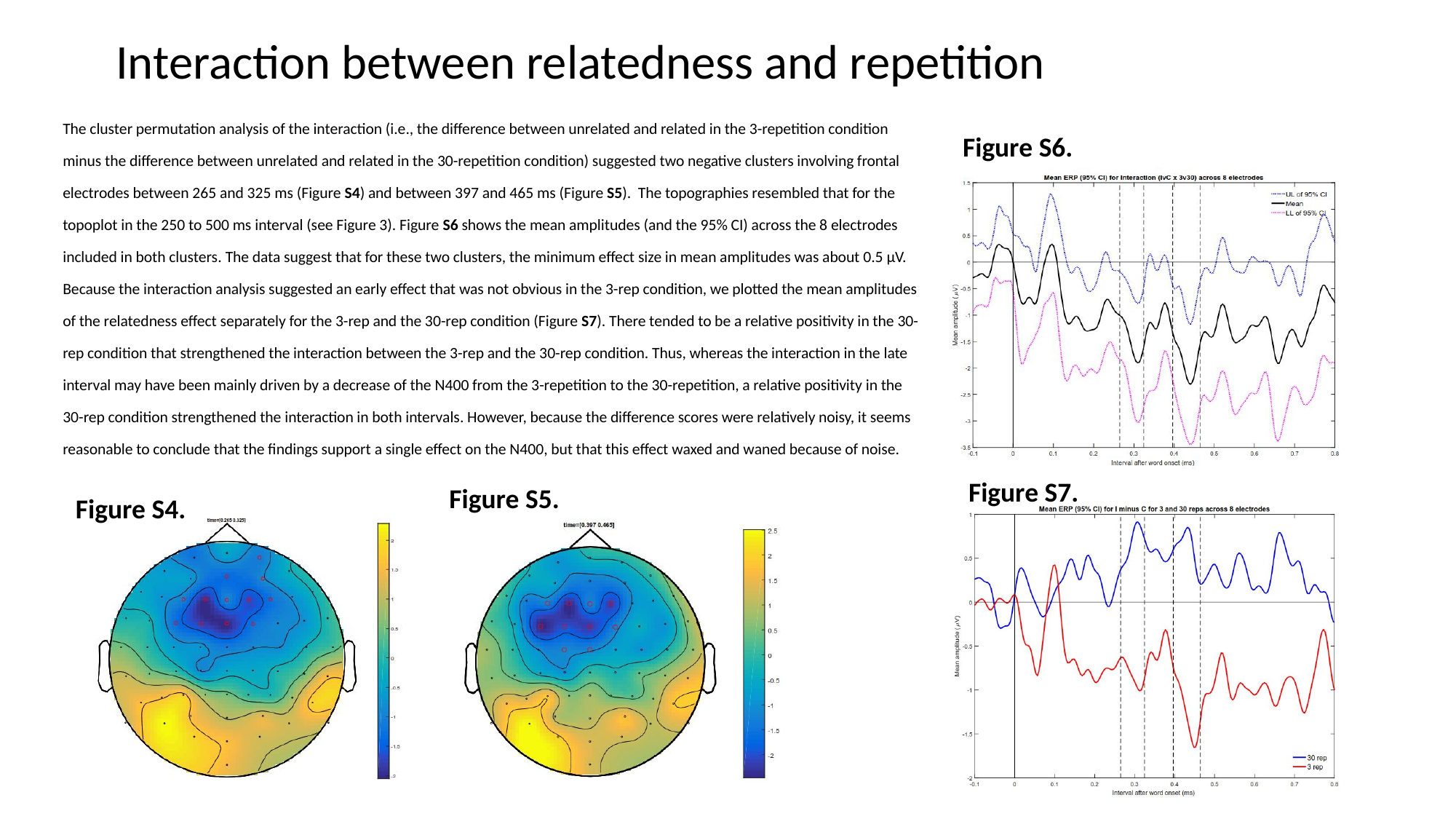

# Interaction between relatedness and repetition
The cluster permutation analysis of the interaction (i.e., the difference between unrelated and related in the 3-repetition condition minus the difference between unrelated and related in the 30-repetition condition) suggested two negative clusters involving frontal electrodes between 265 and 325 ms (Figure S4) and between 397 and 465 ms (Figure S5). The topographies resembled that for the topoplot in the 250 to 500 ms interval (see Figure 3). Figure S6 shows the mean amplitudes (and the 95% CI) across the 8 electrodes included in both clusters. The data suggest that for these two clusters, the minimum effect size in mean amplitudes was about 0.5 µV. Because the interaction analysis suggested an early effect that was not obvious in the 3-rep condition, we plotted the mean amplitudes of the relatedness effect separately for the 3-rep and the 30-rep condition (Figure S7). There tended to be a relative positivity in the 30-rep condition that strengthened the interaction between the 3-rep and the 30-rep condition. Thus, whereas the interaction in the late interval may have been mainly driven by a decrease of the N400 from the 3-repetition to the 30-repetition, a relative positivity in the 30-rep condition strengthened the interaction in both intervals. However, because the difference scores were relatively noisy, it seems reasonable to conclude that the findings support a single effect on the N400, but that this effect waxed and waned because of noise.
Figure S6.
Figure S7.
Figure S5.
Figure S4.
